# Site-specific bacterial chromosome engineering mediated by IntA integrase from *Rhizobium etli*

**DOI:** 10.1186/s12866-016-0755-y

**Published:** 2016-06-29

**Authors:** Rogelio Hernández-Tamayo, Gonzalo Torres-Tejerizo, Susana Brom, David Romero

**Affiliations:** Programa de Ingeniería Genómica, Centro de Ciencias Genómicas, Universidad Nacional Autónoma de México, Apartado Postal 565-A, 62210 Cuernavaca, Morelos Mexico; Departamento de Ciencias Biológicas, Instituto de Biotecnología y Biología Molecular, UNLP, CCT-La Plata-CONICET, Facultad de Ciencias Exactas, Universidad Nacional de La Plata, La Plata, Argentina

**Keywords:** Tyrosine recombinase, Chromosomal integration, Site-specific recombination

## Abstract

**Background:**

The bacterial chromosome may be used to stably maintain foreign DNA in the mega-base range. Integration into the chromosome circumvents issues such as plasmid replication, stability, incompatibility, and copy number variance. The site-specific integrase IntA from *Rhizobium etli* CFN42 catalyzes a direct recombination between two specific DNA sites: *attA* and *attD* (23 bp). This recombination is stable. The aim of this work was to develop a *R. etli* derivative that may be used as recipient for the integration of foreign DNA in the chromosome, adapting the IntA catalyzed site-specific recombination system.

**Results:**

To fulfill our aim, we designed a *Rhizobium etli* CFN42 derivative, containing a “landing pad” (LP) integrated into the chromosome. The LP sector consists of a green fluorescent protein gene under the control of the *lacZ* promoter and a spectinomycin resistance gene. Between the *lacZ* promoter and the GFP gene we inserted an IntA *attachment* site, which does not affect transcription from the *lac* promoter. Also, a mobilizable donor vector was generated, containing an *attA* site and a kanamycin resistance gene; to facilitate insertion of foreign DNA, this vector also contains a multicloning site. There are no promoters flanking the multicloning site. A biparental mating protocol was used to transfer the donor vector into the landing pad strain; insertion of the donor vector into the landing pad sector via IntA-mediated *attA* X *attA* recombination thereby interrupted the expression of the green fluorescent protein, generating site-specific cointegrants. Cointegrants were easily recognized by screening for antibiotic sensitivity and lack of GFP expression, and were obtained with an efficiency of 6.18 %.

**Conclusions:**

Integration of foreign DNA in Rhizobium, lacking any similarity with the genome, can be easily achieved by IntA-mediated recombination. This protocol contains the mating and selection procedures for creating and isolating integrants.

## Background

A critical factor for genome engineering of complex, novel characteristics in bacteria, is the efficient introduction and stabilization of large segments of DNA lacking similarity to the host genome. Plasmid-based strategies are of limited use in this regard, due to problems of plasmid instability and unforeseen consequences generated by copy number variations. Site-specific recombination mediated by integrases can neatly circumvent these problems, providing a means for effective integration of large DNA fragments in specific genomic locations. Tyrosine recombinases are one of the two classes of proteins that catalyze site-specific recombination [1]. Most of the members of this family are highly efficient in promoting recombination, leading to excision, integration or both events, although in the case of bidirectional tyrosine recombinases, with contrasting efficiencies. Unlike homologous recombination, which requires extensive similarity between recombining sequences, tyrosine recombinases catalyze recombination between substrates sharing limited sequence identity. Sequence identity normally spans a short region (8–15 bp) where DNA exchange occurs. The properties of high efficiency and reduced size of target sequences for recombination, make integrases excellent tools for the manipulation of DNA. Integrases are also amenable to a variety of genetic modifications that may enable the production of custom integrases with selectivity for novel target sequences [2].

Systems for chromosomal integration of foreign DNA mediated by tyrosine integrases were devised as early as the 1990s for *Escherichia coli* [3], *Staphylococcus aureus* [4] and *Actinobacteria* [5, 6]. All of them employ special plasmid vectors harboring the corresponding recombinase recognition sequence (*att* or *attachment* site), where foreign DNA can be cloned. Upon introduction into target cells expressing the cognate integrase, site-specific integration occurs *via* the endogenous *attachment* site.

The two most widely used systems for in vivo recombination based on tyrosine recombinases are Cre-loxP and Flp-frt [7]. These systems have a proven efficacy in a variety of biological systems. However, both promote excision more readily than integration. Moreover, given the complex manipulations needed for generation for strains with new features, there is a growing demand of novel systems that employ different recombination systems.

In a previous study we characterized the function of the *Rhizobium etli* IntA site-specific recombinase, through a combination of in vivo and in vitro assays [8]. IntA belongs to the tyrosine-recombinase family. It allows cointegration of plasmids p42a and the symbiotic plasmid via site-specific recombination between *attachment* regions in *R. etli*. The results described previously revealed that IntA is able to catalyze both integration (2.4×10^−5^) and excision (2.1×10^−6^) events in vivo [8]. The recombination requires the presence of a characteristic 23 bp sequence (the *att* sector) harboring two arms of a palindrome plus a divergent central region [8]. The sequence of *attA* (TCCGATAAGCA*T*TACTTATCGGA) differs by a single nucleotide (shown in italics) from *attD*; this difference does not affect recombination efficiency with IntA, since *attA* X *attA*, *attA* X *attD* and *attD* X *attD* recombination are all equally likely [8].

In the present study, we took advantage of the high efficiency and specificity of integration afforded by this system, to construct an integration system for *R. etli* based on site-specific recombination via IntA integrase. This system allows integration of large DNA segments, in a manner independent of homologous recombination, into predefined sectors in the genome of *R. etli.*

## Results and discussion

**Fig. 1:**
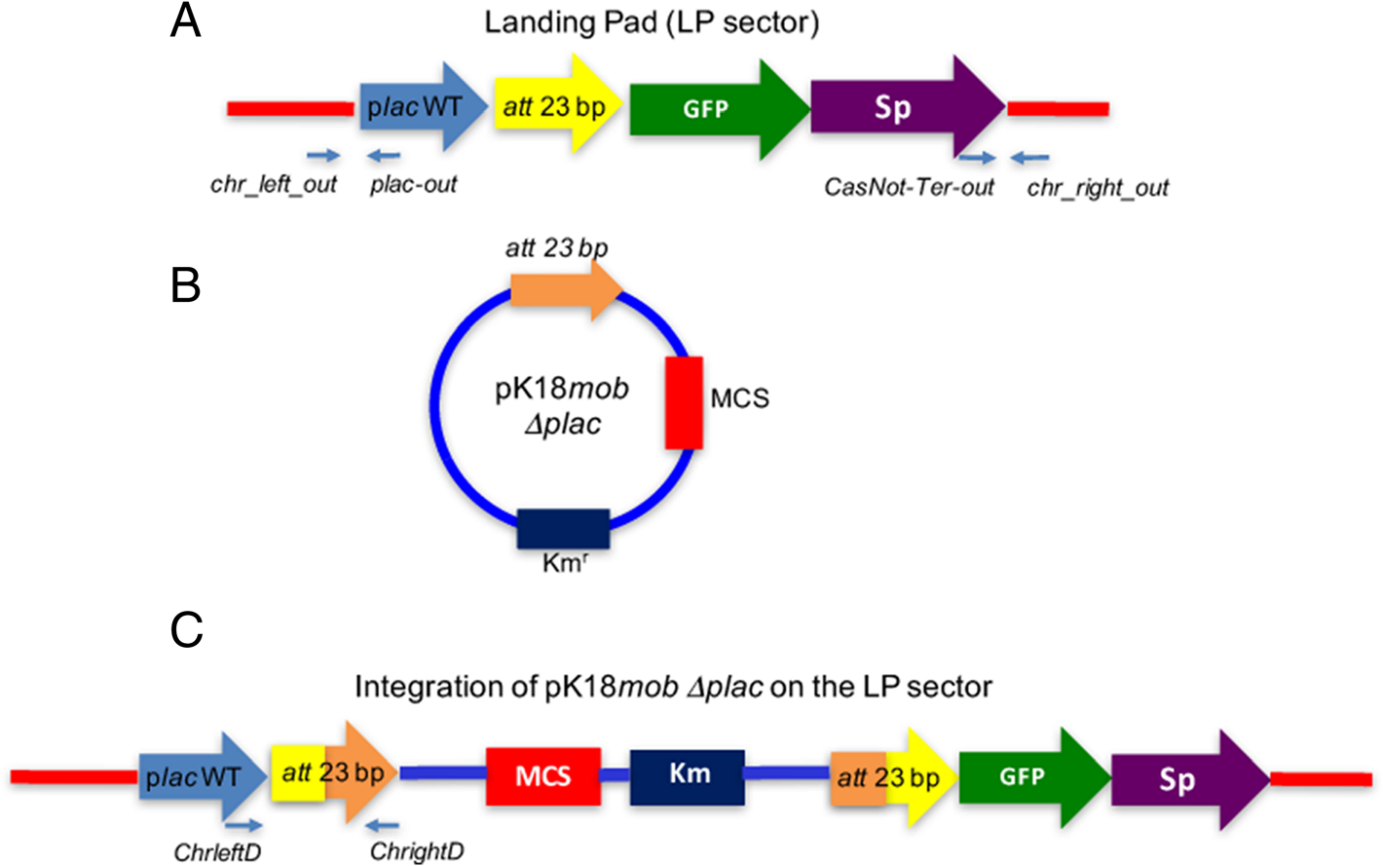
Diagram of the IntA site-specific integration approach. **a** A novel region (the Landing Pad or LP sector) was inserted into the chromosome of *R. etli.* This region comprises a new *attA* region, flanked by a *lac* promoter and a promoterless green fluorescent protein (GFP) gene; and a spectinomycin resistance gene with its own promoter. **b** Mobilizable kanamycin-resistant donor vector (pK18 *mob att Δplac*) containing an *attD* site and a Multi Cloning site (MCS). The p*lac* promoter was removed from the donor vector as described in Methods. **c** Predicted structure of integrants of pK18 *mob att Δplac* into the LP sector. Note that integration of the donor vector by *attA* X *attD* recombination abolishes transcription of the GFP gene, leading to nonfluorescent colonies. In panels **a** and **c**, the location of oligonucleotide primers useful to verify insertion, are indicated as arrows below the appropriate locations

*R. etli* has two IntA-dependent recombination sites, *attA* present in plasmid pRetCFN42a and *attD* on plasmid pRetCFN42d [9]. Aiming to introduce a supernumerary *att* site on the chromosome, we modified a previous construction, that contained a green fluorescent protein gene under the control of the *lacZ* promoter and a spectinomycin resistance gene [10]. This construction was modified by inserting an IntA att site between the *lacZ* promoter and the GFP gene. This insertion does not affect transcription from the *lacZ* promoter (see Methods); the whole region was called the “landing pad” (LP) sector (Fig. 1a). For insertion of the LP sector into the chromosome, a region was chosen, where insertion of additional sequences most likely does not interfere with essential activities of *Rhizobium etli*. Previous genomic analyses revealed a non-coding region in the chromosome, located between bases 1108084 and 1108560; this region is flanked by converging transcription units corresponding to loci RHE_CH01055 and RHE_CH01056 [11]. To facilitate insertion of the LP sector, a chromosomal segment (477 bp, corresponding to the non-coding region) was amplified using primers (Table 1). Insertion of the LP sector in the middle of this chromosomal segment (see Methods) provided extensions of 221 bp (5′side) and 249 bp (3′side), homologous to the chromosome. Insertion of the LP sector into the chromosomal region was readily done by allelic exchange (see Methods and Fig. 1a).

**Table 1:** Oligonucleotides used in this work

Name	Sequence ^a^	Genome location ^b^	Source or reference
*chr_left_in_hind*	AAAA*AAGCT*TTCCCGGCTCCGACAG	1108084 Chr	This work
*chr_right_in_eco*	AAAA*GAATTC*CCGGTGTCTGCTTCCA	1108560 Chr	This work
*chr_left_out*	CGGAACACCGGATCTCA	1107995 Chr	This work
*chr_right_out*	CGTGCCCGCTTTTGTC	1108840 Chr	This work
*Plac-out*	CGCGGAACCCCTATTTG		10
*CasNot-Ter-out*	CCGTCTGTGATGGCTTCC		10
*UpattA1*	CT*GGATCC*CAAAGGTCGCTCCTGAAT	2252 p42a	8
*LwattA1*	C*GAATTC*ATTTTCCCGAGAATAACATG	2687 p42a	8
*Lw23bp*	CGGATCCTCCGATAAGCATTACTTATCGGACTTAAGT	5765 p42d	8
*Up23bp*	CGGATCCTCCGATAAGCATTACTTATCGGACTTAAGT	5743 p42d	8
*M13 reverse*	CAGGAAACAGCTATGAC		ThermoFisher Scientific
*LwattD1*	C*GAATTC*CGGCGTTTGCATCTCGTTA	5875 p42d	8
*ChrleftD*	CT*GGATCC*TCCGATAAGTAA		This work
*ChrightD*	C*GAATTC*TGATTTGCGGGCG		This work
*G18lw*	C*GGATCC*TCCGATAAGCATTACTTATCGGACTTAAGT	attG18mob	This work
*G18up*	GCCTAGGAGGCTATTCGTAATGAATAGCCT*GAATTC*A	attG18mob	This work
*Link-PstxNot*	AGCGGCCGCTTGCA	NA	

^a^All oligonucleotides are shown in the 5′ to 3′ direction. Built-in restriction sites, depicted in italics, are *Eco*RI (GAATTC) *Bam*HI (GGATCC) *Hind*III (AAGCTT) *Not*I (GCGGCCGC)

^b^The location is indicated by the first 5′ nucleotide and the replicon where the sequence is located. Accession numbers are p42a (NC_007762), p42d (NC_004041), Chr (NC_007761) of *R. etli.* NA, not applicable (NA)

**Fig. 2:**
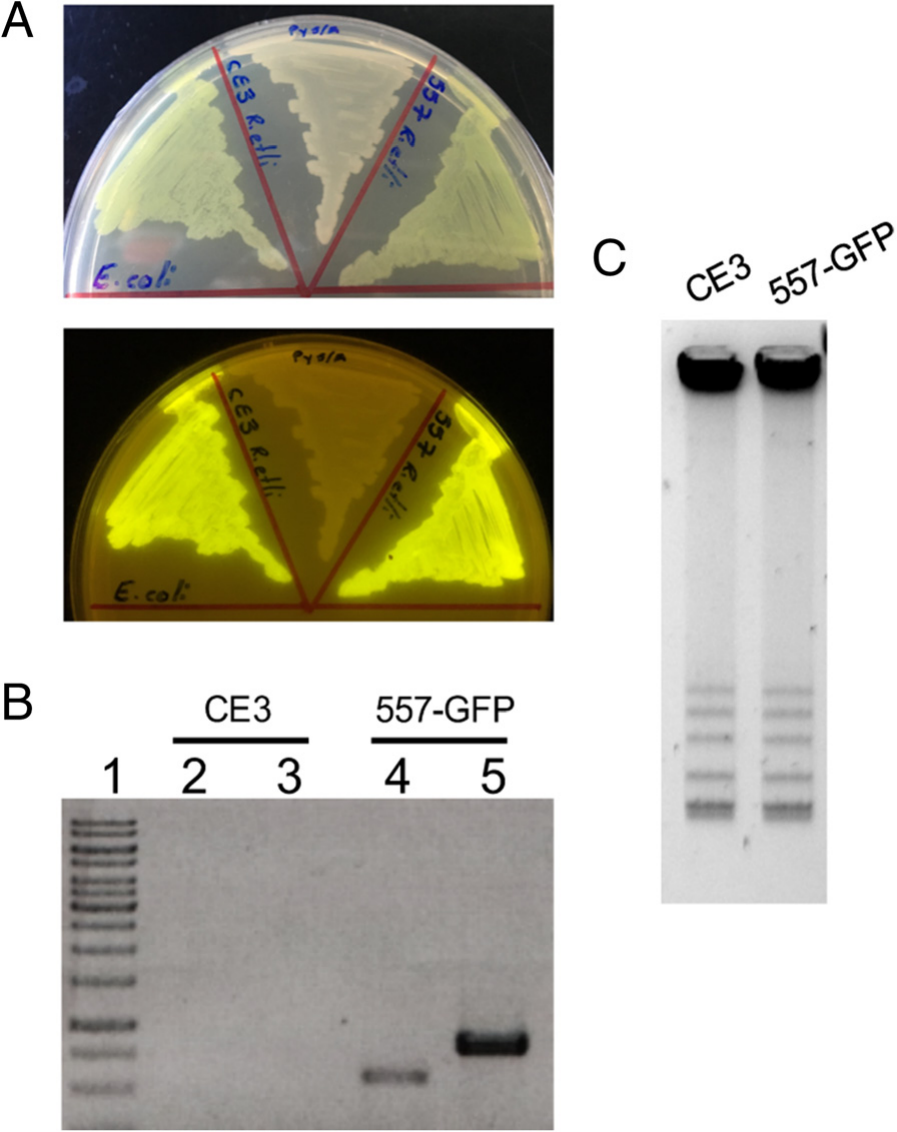
Characterization of the *R. etli* LP strain (557). **a** Visualization of selected strains under white light (top) and UV light (bottom). Strains, in a clockwise order were: *E. coli* harboring plasmid pRG11 (Landing Pad); *R. etli* wild type (CE3); *R. etli* 557 (LP strain). **b** PCR reactions verifying that integration occurred in the desired chromosomal site. Lanes 2 and 4 are PCR reactions with primers *chr_left_out* and *Plac-out*, while lanes 3 and 5 contain PCR reactions with primers *CasNot-Ter-out* and *chr_right_out.* The strains analyzed are indicated in the top of the panel. **c** Plasmid profile of the same strains analyzed in (**a**) and (**b**)

To verify that insertion of the LP sector had occurred in the appropriate chromosomal region, fluorescent derivatives (Fig. 2a) were subjected to PCR amplifications with primer pairs *chr_left_out-Plac-out* and *chr_right_out-CasNot-Ter-out*. For both reactions, one of the members of the primer pair is complementary to sequences of the LP sector, while the other is complementary to the neighboring genomic region. As expected, PCR products were detected with both primer pairs (Fig. 2b), ascertaining that the LP sector was inserted in the appropriate chromosomal region. To verify the absence of gross modifications in the plasmid content of the strain carrying the LP sector, plasmid size and content were verified by the Eckhardt in-gel lysis technique [12] as modified by Hynes [13]. No alterations were detected in the plasmid profile (Fig. 2c).

To evaluate if the LP sector can be used as an acceptor of IntA-mediated in vivo recombination, we employed plasmid pK18 *mob att Δplac* (see Methods and Fig. 1b). This plasmid is a modification of the suicide vector pK18 *mob* containing an *attA* site, a kanamycin resistance gene, and lacking the *placZ* promoter; it also harbors a MCS, where defined segments can be easily introduced. In all our experiments the IntA activity was provided from the *intA* gene encoded in plasmid pRetCFN42a, present in the LP strain. Plasmid pK18 *mob att Δplac* was mobilized by biparental matings, from an *E. coli* donor to the *R. etli* LP strain. Upon entrance of the plasmid into the LP strain, IntA-mediated recombination may occur with any of three targets: the *attA* located in plasmid pRetCFN42a, the *attD* from plasmid pRetCFN42d, or the *attA* present in the chromosomal LP sector. Insertions of pK18 *mob att Δplac* in the chromosomal LP sector disrupt transcription of the GFP gene coming from the *lacZ* promoter (Fig. 1c). Thus, insertions in the LP sector can be easily detected by a simple screen for kanamycin and spectinomycin resistant, nonfluorescent derivatives.

**Fig. 3:**
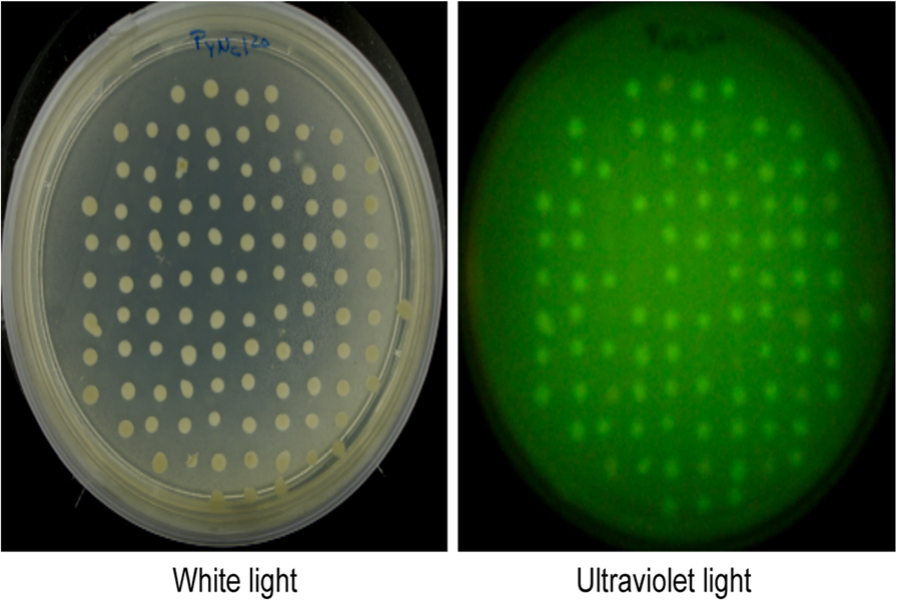
Isolation of integrants. Visualization of random integrants resulting from integration of pK18 *mob att Δplac* into *R. etli* 557. Note the presence of a relatively high frequency of nonfluorescent colonies, indicating integration of pK18 *mob att Δplac* into the LP sector

Upon conjugative transfer of pK18 *mob att Δplac* to the *R. etli* LP strain, kanamycin resistant integrants were detected at a frequency of 1.4×10−4 per recipient cell. Individual integrants were picked and visualized under UV light; representative results are shown in Fig. 3. A total of 1019 integrants (coming from five independent experiments) were analyzed for GFP expression; of these, 63 (6.18 %) were nonfluorescent, thus indicating insertions in the LP sector. The remaining integrants were probably inserted in the *att* sites located on pRetCFN42a or pRetCFN42d. Augmented integration into plasmids in comparison to the chromosome was expected, given the higher copy number of these replicons, estimated at two for pRetCFN42d and four for pRetCFN42a, with respect to the chromosome (D. Romero, unpublished data).

**Fig. 4:**
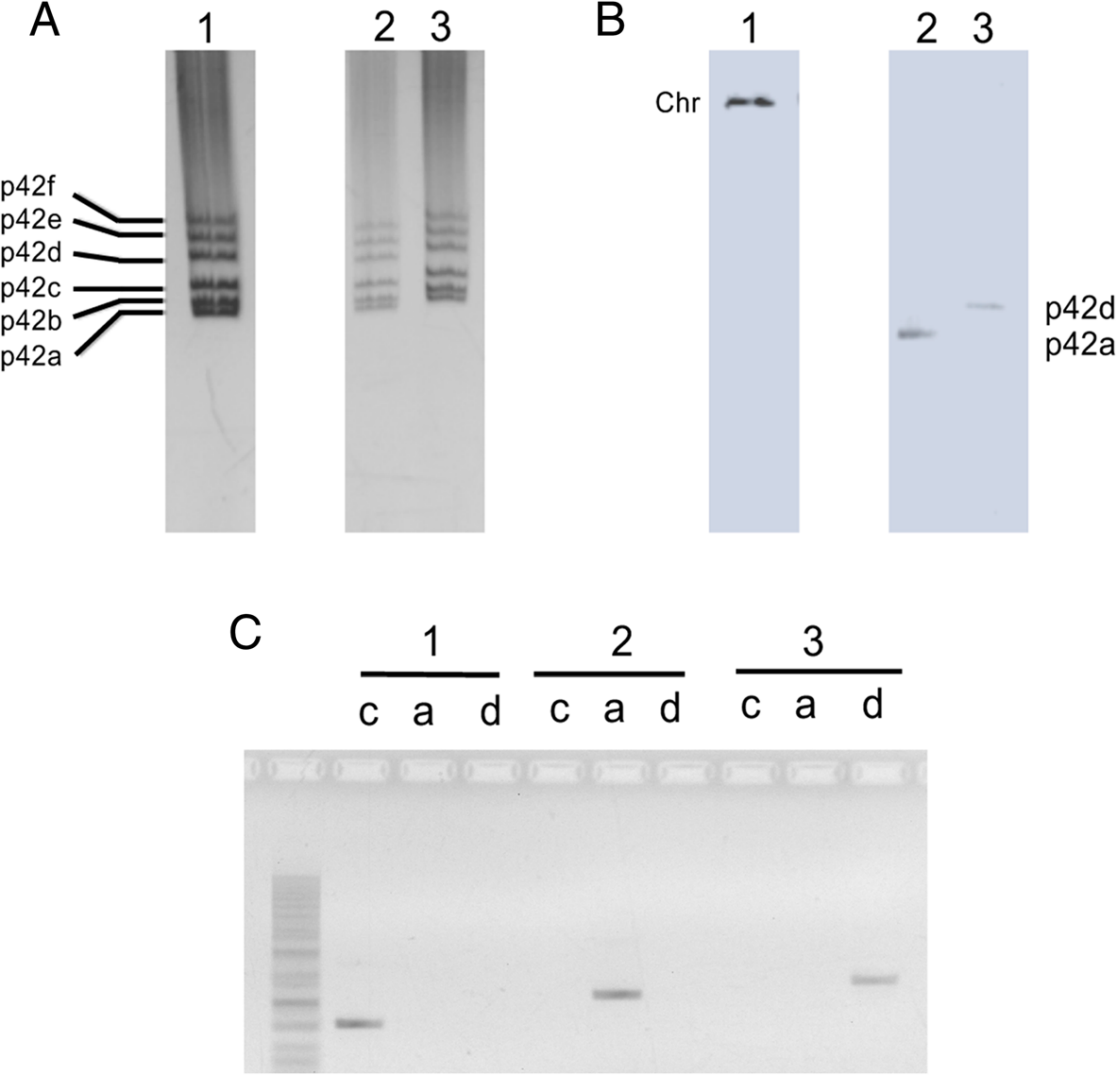
Analyses of selected cointegrates. All panels: lanes or blocks marked 1 correspond to a nonfluorescent kanamycin resistant cointegrate, while lanes or blocks 2 and 3 correspond to different fluorescent kanamycin resistant cointegrates. **a** Plasmid profiles of selected strains, stained with ethidium bromide. Plasmid identification is depicted at the left of the panel. **b** Southern blots of the corresponding plasmid profile, revealed by autoradiography, using ^32^P-labelled pK18*mob* as probe. Chr, chromosome. **c** PCR products of three different strains with a set of primers that distinguish among insertions for the LP sector in the chromosome (lanes c, primers *ChrleftD* and *ChrightD*), or pRetCFN42a (lanes a, primers *LwattA1* and M13 reverse) and pRetCFN42d (lanes d, primers *LwattD1* and M13 reverse)

To verify the site of insertion of each class of integrants, both, nonfluorescent kanamycin and spectinomycin resistant derivatives, as well as fluorescent, kanamycin and spectinomycin resistant integrants, were subjected to PCR analysis with a set of primers that distinguish among insertions in the LP sector (*ChrleftD* and *ChrightD*), pRetCFN42a (*LwattA1* and M13 reverse*)*, or pRetCFN42d (*LwattD1* and M13 reverse). As shown in Fig. 4, nonfluorescent, kanamycin and spectinomycin resistant derivatives rendered a PCR product with primers specific for the LP sector, but not with primers specific for pRetCFN42a or pRetCFN42d (Fig. 4c). In contrast, fluorescent, kanamycin and spectinomycin resistant integrants occurred either in pRetCFN42a or pRetCFN42d, depending of the integrant. Location of the insertions was further verified using Southern blot hybridizations of the plasmid profiles with pK18 *mob* as probe (Fig. 4a and b). The six nonfluorescent, spectinomycin resistant derivatives analyzed in this way presented insertion on the LP sector; from five fluorescent, spectinomycin resistant integrants that were screened, three revealed integration of pK18 *mob att Δplac* on pRetCFN42a, while in the remaining two, insertion occurred on pRetCFN42d.

To evaluate the stability of integrants of pK18 *mob att Δplac* in the LP sector, one integrant was serially subcultured in the absence of antibiotics, for a period corresponding to 21 generations. Dilutions were plated on solid media without antibiotics. A total of 10 000 colonies were screened for GFP expression, interestingly, all of them remained nonfluorescent.

**Fig. 5:**
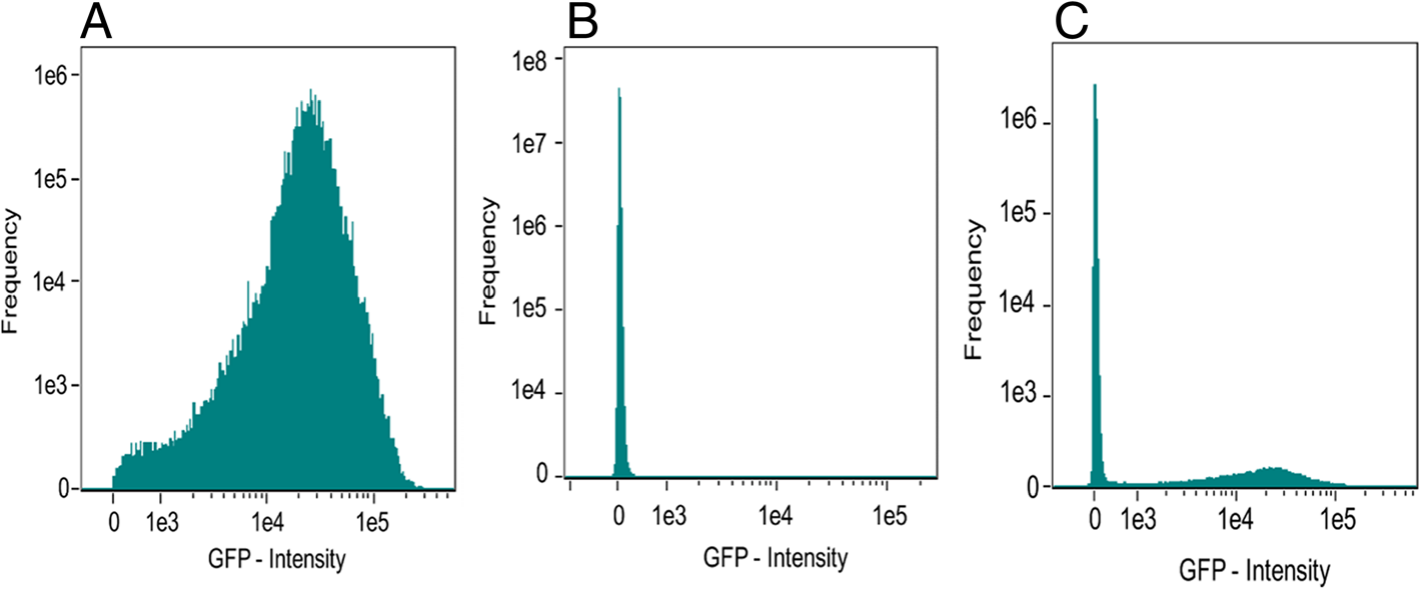
Flow cytometry analyses. For each sample, data from a minimum of 10^7^ cells serially subcultured in the absence of antibiotics, for a period representing 21 generations were collected using ImageStream X imaging flow cytometer and analyzed using IDEAS 5.0 software. **a** cells with insertion of the LP sector on the chromosome display strong fluorescence. **b** cells with pK18 *mob att Δplac* inserted in the LP sector. **c** Mixture of cells corresponding to integration of pK18 *mob att Δplac* in the LP sector (10^7^ cells) and the strain harboring the LP sector (10^3^ cells)

To further explore the stability of integrants on the LP sector, flow cytometry analyses were carried out. In these experiments, 10^7^ cells derived from serial subcultures in the absence of antibiotics (21 generations) were screened for fluorescence, thus constituting a more robust assay. As shown in Fig. 5a, most of the cells corresponding to the insertion of the LP sector on the chromosome display strong fluorescence. Only about 100 cells (1 × 10^−5^) in this population failed to display fluorescence, but these appear to be mainly young cells. In contrast, in a population of 10^7^ cells containing the pK18 *mob att Δplac* integrated in the LP sector, all the cells failed to display fluorescence, (Fig. 5b). To verify that this assay can reliably identify fluorescent cells out of a large population of nonfluorescent cells, 10^7^ cells with pK18 *mob att Δplac* integrated in the LP sector were mixed with 10^3^ fluorescent cells corresponding to the strain harboring the LP sector. As shown in Fig. 5c, flow cytometry analysis clearly reveals two populations differing in fluorescence, in the expected proportions. Overall, the results presented above confirm that the insertions on the LP sector are highly stable.

After demonstrating that integration may occur on the LP sector, we then asked if integration on the LP sector still leaves it susceptible to further integration events. Integrants on the LP sector possess two *att* sites separated by the inserted DNA. These integrants are nonfluorescent due to the integration of sequences lacking an active promoter for transcription of the GFP gene. We reasoned that further integration of a sequence harboring an active promoter on the rightmost *att* site (i. e. the one closer to the GFP gene) should restore GFP production.

**Fig. 6:**
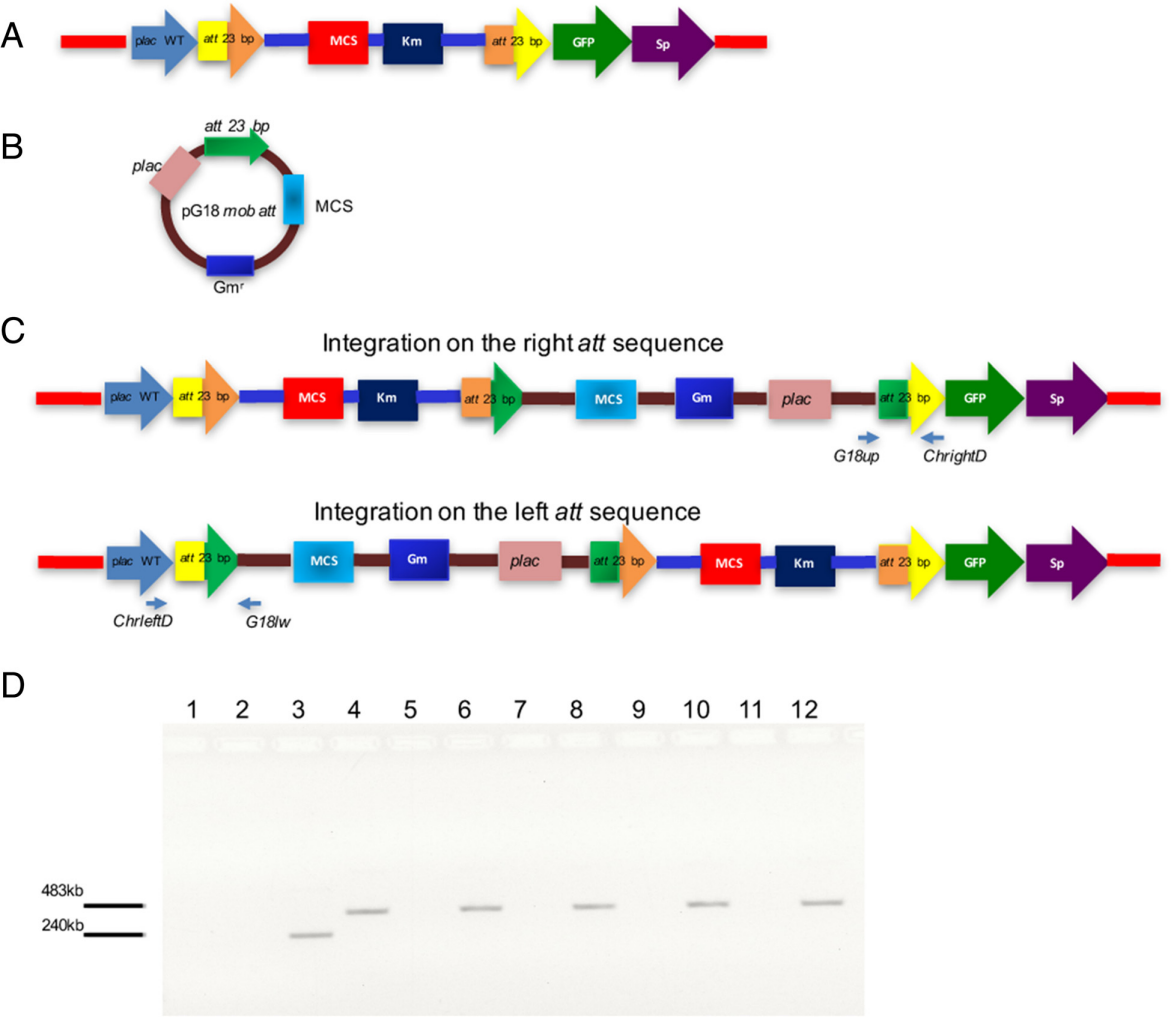
Integrants on the LP sector may acquire additional sequences. **a** Structure of integrants carrying pK18 *mob att Δplac* in the LP sector. Note that integration generates a duplication of att sequences. **b** Mobilizable gentamicin resistant donor vector (pG18 *mob att*) containing an *attA* site, a *plac* promoter and a Multi Cloning site (MCS). **c** Predicted structure of integrants of pG18 *mob att* into the integrant shown in (**a**). Notice that integration of the donor vector into the rightmost *att* sequence places a *plac* promoter in proximity to the GFP gene. **d** Specific PCR assays demonstrating the occurrence of additional insertions within the LP sector. Control strain 557 (lanes 1 and 2); five strains with additional insertions, arranged in pairs (lanes 3 to 12). Lanes with odd numbers correspond to amplification with a set of primers revealing integration on the left *att* sequence (*ChrleftD*-*G18lw*) and even numbers correspond to amplification with primers complementary to the right side (*ChrightD-G18up*), of the LP sector. Note that most of the strains harbor additional insertions on the rightmost *att* site, while the strain depicted in lanes 3 and 4 carry insertions on both the left and the right *att* sites

To explore this, we took advantage of the previously built *Bordetella* suicide plasmid pG18*mob* (Gm^r^) [14]. This plasmid lacks any detectable sequence similarity with pK18 *mob att Δplac*, other than the *mob* segment, and it possesses a p*lacZ* promoter adjacent to the MCS sequence. Into the MCS sequence of this plasmid, an *att* fragment was cloned as an *EcoR*I-*Sal*I fragment (see Methods and Fig. 6b). After conjugative transfer of pG18 *mob att* to the *R. etli* LP strain that already contained an integrated pK18 *mob att Δplac*, Km^r^ Gm^r^ integrants were detected at a frequency of 1.8 × 10^−4^ per recipient cell. A total of 261 integrants (from two independent experiments) were analyzed for GFP expression; 8 (3.06 %) of them recovered fluorescence production. These results are compatible with further insertions on the LP sector. These integrations may have occurred on the *att* sequence located either at the left side or the right side of the LP sector (Fig. 6c). We reasoned that insertions on the right side were most likely, since they place a *placZ* promoter in proximity to the GFP gene, thus explaining their fluorescent nature.

To verify the location of additional insertions within the LP sector, specific PCR assays were set up. These PCR assays comprised two sets of primers; the first one included a primer complementary to the left side of the LP sector (*ChrleftD*) and the other matched the sequence of pG18 *mob att* (*G18lw*) while the second pair had a primer complementary to the right side of the LP sector (*ChrightD*) and another to pG18 *mob att* (*G18up*). Amplification with the first set of primers revealed integration on the left *att* sequence, while PCR products obtained with the second set reveals integration on the right *att* sequence (Fig. 6c). As expected, four of the integrants characterized revealed amplification with the second set of primers but not with the first set, indicating that integration indeed occurred on the rightmost *att* site (Fig. 6d). Interestingly, one of the fluorescent derivatives gave positive amplification with both primer sets, indicating the possibility of multiple integration events on the LP sector (Fig. 6d).

## Conclusions

As shown in this work, site-specific bacterial chromosome engineering mediated by IntA allows the efficient integration of sequences lacking extensive homology to the host genome, other than the *att* sequence. Use of this approach requires a specially-constructed strain, harboring the so-called Landing Pad, containing a single *att* region, as well as GFP to allow easy identification of strains harboring the desired integration. The size of the construct to be integrated is limited only by the size of DNA that can be successfully cloned into the donor vector via ligation. Transfer of foreign DNA into the LP strain is done by conjugation, thus circumventing the need for DNA purification and transformation, making it a very robust approach. Under appropriate conditions, integrants were derived at a high frequency (10^−4^) compared to the frequency of double crossover recombination (10^−6^). The integrated DNA is flanked by copies of the *att* sequences, which are potential targets for IntA-mediated excision. Nevertheless, integrants are very stable, due to the low frequency of excision, evaluated previously at 2.1×10^−6^ [8]. In fact, in the experiments reported here, no IntA-mediated excision events were detected. The finding that the *att* sequences added by integration are still active for recombination, open up the possibility of larger, more complex alternatives of strain construction in a sequential way.

Although this is not the first system described that allows integration mediated by site-specific recombination, it offers some advantages compared to other systems. For instance, recombineering via λ-red, a phage based system, requires larger homologous segments, and has limited effectiveness in bacteria other than *E. coli* [15] The ΦC31 integrase has a different recombination target than IntA, and it has demonstrated efficient activity in disparate hosts, including *Sinorhizobium meliloti* [16]. IntA has the advantages of efficiency and modularity, given that any donor cassette can potentially be integrated into any LP-strain. In this regard, several rhizobial species possess IntA-homologs, with conceivably different specificities. Ideally, the IntA system may be combined with other site-specific systems with different specificity, to facilitate bacterial genome engineering.

## Methods

### Bacterial strains, plasmids and growth conditions

*Rhizobium* strains were grown at 30 °C in PY (peptone-yeast extract) medium [17]. *Escherichia coli* strains were grown at 37 °C in LB (Luria-Bertani) medium [18]. When needed, antibiotics were added at the following concentrations (in micrograms per milliliter): nalidixic acid, 20; spectinomycin, 100; kanamycin, 30*.*

### Biparental mating

Plasmid transfer from *E. coli* to *Rhizobium* was done by biparental mating, using *E. coli* S17.1 harboring the suicide vector (pK18 *mob att Δplac*) as a donor and *R. etli* 557 (LP-strain) as recipient, the strains were grown in liquid medium to stationary phase, washed twice with PY medium, mixed in a donor/recipient ratio of 1:2 on PY plates, and incubated at 30 °C overnight. After incubation, cells were resuspended in MgSO_4_ 10 mM-Tween 0.01 %, serially diluted and plated on solid PY medium containing nalidixic acid, spectinomycin and kanamycin. Cointegration frequencies were evaluated independently at least thrice, and are expressed as number of transconjugants per recipient cell. For evaluation of GFP production, colonies were visualized under ultraviolet light (425 nm).

### Molecular and microbiological procedures

Basic DNA manipulations and molecular techniques were done using established procedures [19]. Extraction of DNA from agarose gels was done with a GeneJET extraction kit (Fermentas); plasmids were isolated with high pure plasmid isolation kit (Roche). All oligonucleotides used were synthesized at the Unidad de Síntesis of the Instituto de Biotecnología, Universidad Nacional Autónoma de México; all PCR amplifications were carried out using High Fidelity *Taq* polymerase (Invitrogen). Amplification protocols consisted of 30 cycles of 1 min at 94 °C, 1 min at variable temperature (depending on the primer combination), and 1 to 3 min at 68 °C. After amplification, PCR products were extracted with phenol and precipitated with ethanol. The DNAs were resuspended in Tris-EDTA buffer and digested with the appropriate restriction enzyme(s) to generate the required ends in the fragments. The DNA fragments were purified before cloning by isolating them from the agarose gel. For ligations, T4 polynucleotide ligase (Fermentas) was used. Plasmid transformation of *E. coli* was done using CaCl_2_-competent cells. All plasmid constructions were verified by restriction analysis and PCR and, in most of the cases, by DNA sequencing.

### Plasmid construction

To generate the LP sector, a cassette containing a green fluorescent protein gene under the control of the *lacZ* promoter and a spectinomycin resistance gene [10] was modified by the introduction of an *att* site. To do that, the *attA* site was amplified from *R. etli* genomic DNA using primers *UpattA1* and *LwattA1*, gel-purified and restricted with *EcoR*I and *Bam*HI and ligated into similarly restricted pCR2.1^®^-TOPO^®^ TA vector (Thermo Fisher Scientific) From the resulting plasmid, the *attA* sequence was excised by digestion with *Xba*I and ligated into *Xba*I-digested pGX534 [10], giving rise to plasmid pRG10. In this plasmid, the *lacZ* promoter and the GFP gene are separated by an *att* sequence. To facilitate insertion of the LP sector in the chromosome, a 477 bp chromosomal segment (from nucleotide 1108084 to nucleotide 1108560, corresponding to the non-coding region) was amplified using primers *chr_left_in_hind* and *chr_right_in_eco* (Table 1). This PCR fragment was digested with *Hind*III and *Eco*RI and cloned into similarly digested pK18*mob-sacB* [20] yielding pGX117. To substitute a single *Pst*I site present within the non-coding region with a *Not*I site, pGX117 was digested with *Pst*I and ligated to oligonucleotide Link-*Pst*x*Not*, giving rise to plasmid pGX191.

To generate a chromosomal entry vector for the LP sector, plasmid pRG10 was digested with *Not*I (thus excising a *Not*I cassette harboring the LP sector), and the resulting fragment was cloned into *Not*I digested pGX191. The resulting chromosomal entry vector for the LP sector was called pRG11.

To generate a plasmid useful for integration in the LP sector, a derivative of plasmid pK18 *mob* [20], harboring an *attD* sequence and lacking the native plac promoter was constructed. To do that, two complementary oligonucleotides (Lw23bp and Up23bp, Table 1), containing the *attD* sequence, were heat-denatured and reannealed, and the resulting double-stranded fragment was restricted with *BamHI*. This product was ligated into similarly restricted pK18 *mob*, giving rise to pK18 *mob att*. To remove a 160 bp segment containing the *lac* promoter, pK18 *mob att* was restricted with *AseI* and *EcoRI*, ends were filled-in with Klenow polymerase and the plasmid was religated in a blunt-end ligation, resulting in plasmid pK18 *mob att Δplac* (3.66 kb).

A separate vector, useful to investigate the possibility of repeated insertions into an already occupied LP sector (plasmid pG18 *mob att*) was also constructed. To this end, the *attA* site was amplified from *R. etli* genomic DNA using primers *UpattA1* and *LwattA1*, gel-purified and restricted with *EcoR*I and *Bam*HI and ligated into similarly restricted pG18 *mob* [14], thus generating pG18 *mob att* (2.88 kb)*.*

### Plasmid profiles and hybridization

*Rhizobium* plasmids were visualized by the Eckhardt procedure [12]. Gels were transferred onto Hybond N^+^ membranes (Amersham) using the manufacturer’s protocol and cross-linked using a UV cross linker unit (Stratagene). Hybridizations were performed overnight using α^32^P-dCTP-labelled probes (Megaprime kit; Amersham) under high-stringency conditions (65 °C in rapid-Hyb buffer, Amersham). Hybridization signals were detected with a PhosphorImager (Molecular Dynamics).

### Flow cytometry

For determination of stability of the integrated DNA by flow cytometry, cells from the desired strain were subjected to three serial overnight cultures in the absence of antibiotics (representing about 21 generations) and analyzed by flow cytometry. Cells were analyzed in the ImageStream X imaging flow cytometer (Amnis Corporation, Seattle, WA) using the 488 nm solid-state laser with appropriate compensation controls and settings. Data were acquired using INSPIRE acquisition software and the 60X objective lens on a low speed setting. Data from a minimum of 10^7^ cells were collected for each sample and analyzed using IDEAS 5.0 software. The instrument and INSPIRE software were set up as follows: Channel 01 (bright field), channel 02 (green fluorescence) and channel 12 (scattering channel). Magnification was 60X, providing a pixel size of 0.3 μm2 and the lasers 488 and 745 activated for fluorescence and side-scatter, respectively. The flow rate was set to low speed/high sensitivity and stream alignment was adjusted where necessary. Focused images of single cells were initially identified from the brightfield images taken by the imaging flow cytometer by using the manufacturer’s recommended analysis algorithm, the gradient root mean square feature. To reduce artifacts and background noise, we used the default mask of the IDEAS 5.0 software called M01 and an investigator-defined mask, which was composed of a combination of mask features (system 50, erode 2, range 300–2,000). Areas of interest smaller than 300 pixels were deselected. Images that still had a spot count of two or more, were excluded and designated as “doublets.”

## Abbreviations

°C, celsius degree; *att*, attachment site; *attA*, attachment site plasmid a; *attD*, attachment site plasmid d; Bp, base pairs; Chr, chromosome; DNA, deoxyribonucleic acid; EDTA, ethylenediaminetetraacetic acid; GFP, green fluorescent protein; Gm^r^, gentamicin resistant; LB, luria-bertani; LP, landing pad; MCS, multi cloning site; Nm, nanometer; PCR, polymerase chain reaction; PY, peptone-yeast extract; UV, ultraviolet.
